# The common cold, allergy, and cancer.

**DOI:** 10.1038/bjc.1986.161

**Published:** 1986-07

**Authors:** C. Chilvers, B. Johnson, S. Leach, C. Taylor, E. Vigar


*Br. J. Cancer* (1986), **54**, 123–126

### Short Communication

### The common cold, allergy, and cancer

C. Chilvers, B. Johnson, S. Leach, C. Taylor & E. Vigar

*Section of Epidemiology, Institute of Cancer Research, Block D, Clifton Avenue, Sutton, Surrey, UK.*

During 1982, one of us (EV) observed in the course of health visiting that patients with cancer had common colds less frequently than patients with other diseases. A small case-control study of the relationship between history of common colds and cancer was carried out by this same observer and the results confirmed her previous observation. More recently Remy *et al.* (1983) carried out a similar study; from data given in their Figure 1, the relative risk of cancer is approximately 6 in individuals having an average of less than one cold per year over the 10 years before diagnosis compared to those having one or more colds per year. We have now undertaken a hospital based case-control study to test these findings.

Cases were identified by one of two research assistants who visited every co-operating ward at St Helier Hospital, Carshalton, each week. Confirmed and possible cases of cancer were selected if they were aged between 46 and 75, had no previous history of cancer or psychiatric disorder, and were well enough to be interviewed. Lung cancer was excluded because of the overwhelming smoking association. Similar age and sex distributions for cases and controls were obtained by matching possible controls to possible cases. Two types of control were selected, the first with a serious (i.e. potentially life-threatening) illness and the second with a diagnosis of a more minor nature, for example varicose veins or inguinal hernia. Patients admitted with respiratory diseases or with a previous malignancy (except non-melanomatous skin cancer) were excluded from the control group. A list of patients to be interviewed was given to the interviewer on the day of selection; cases and controls were not identified as such. The interviewer attempted to interview every listed patient before they left hospital using a standard questionnaire. After interview the case notes were checked for diagnosis and history of previous malignancy.

We thought it important that the interviewer should be 'blind' to the primary purpose of the study. For this reason we wished to include questions additional to those concerning colds in the questionnaire. History of allergy seemed a good choice for inclusion because of the contradictory results obtained from earlier studies (Logan & Saker, 1955; Fisherman, 1960; Mackay, 1966; McKee *et al.*, 1967; Ure, 1969; Shapiro *et al.*, 1971; Gabriel *et al.*, 1972; Meers, 1973; Alderson, 1974; Polednak, 1975; Allegra *et al.*, 1976; Robinette & Fraumeni, 1978). Headache and migraine were included because we thought it unlikely that they would be related to development of cancer and would act as a check on the validity of our results. The interview lasted approximately ten minutes and also included questions on smoking habits and demographic details. Statistical analysis was carried out using standard methods (Breslow & Day, 1980). Permission to carry out this study was obtained from the District Ethical Sub-committee.

A total of 120 cases, 151 controls with serious conditions (group A) and 88 with non-serious conditions (group B) were interviewed (Table I). Seven patients (2%) refused to be interviewed. The imbalance in numbers between cases and serious controls arose because the diagnosis was often uncertain when the case and control selection was made and was subsequently changed on review. A control with a relatively trivial complaint could not be found for every case. The characteristics of the three groups are shown in Table II; the groups are similar with respect to demographic characteristics and smoking habit. Relative risks of cancer in relation to history of common colds and history of other ailments are shown in Table III; the 95% confidence intervals indicate that none of the odds ratios differs significantly from unity. In Table III the result reported for common colds is restricted to patients who reported that they had had a similar number of colds each year for at least 10 years prior to diagnosis. The cut-off point chosen (less than one cold per year *versus* one or more per year) is identical to that used by Remy *et al.* (1983). Additional analyses used a narrower grouping of the numbers of colds reported (less than one, one, and 2 or more per year) to test for a trend in relative risks. There was no evidence of such a trend. The mean number of colds per year reported by cases, control group A and control group B was 1.2, 1.3 and 1.4 respectively. All

Correspondence: C. Chilvers.
Received 2 January 1986; and in revised form 13 March 1986.

© The Macmillan Press Ltd., 1986

**Table I** Sites of cancer and control diagnosis

| *Sites of cancer* | | *Control group A* | | *Control group B* | |
|---|---|---|---|---|---|
| G.I. tract | 58 | Cardiovascular | 22 | Circulatory | 19 |
| Female breast | 12 | Cerebrovascular | 14 | Gastrointestinal | 22 |
| Female reproductive organs | 7 | Other vascular | 17 | Genitourinary | 29 |
| Prostate | 10 | Gastrointestinal | 31 | Other | 18 |
| Urinary tract | 15 | Genitourinary | 29 | | |
| Lymphatic and haematopoietic | 10 | Fractures & injury | 15 | | 88 |
| Other | 8 | Other | 23 | | |
| | 120 | | 151 | | |

**Table II** Characteristics of case and control groups

| | *Cases* *n=120* | *Control A* *n=151* | *Control B* *n=88* |
|---|---|---|---|
| Age (yrs): mean (s.d) | 64.8 (7.4) | 63.7 (8.0) | 62.4 (8.6) |
| Sex: % male | 50.8% | 44.4% | 50.0% |
| Martial status: | | | |
| Single | 8.3% | 9.9% | 3.4% |
| Married | 66.7% | 67.5% | 72.7% |
| W+D+Sep[a] | 25.0% | 22.5% | 23.9% |
| Social class: | | | |
| % non-manual | 46.6% | 37.5% | 45.3% |
| Smoking: | | | |
| Current smoker | 30.0% | 37.1% | 27.3% |
| Ex-smoker | 38.3% | 31.1% | 39.8% |
| Non-smoker | 31.7% | 31.8% | 33.0% |
| % with children aged up to 15 living in household | 2.5% | 5.2% | 8.0% |

[a]Widowed, Divorced or Separated.

**Table III** Relative risk of cancer in relation to history of common colds and other ailments

| | *Positive history (%)* | | *Odds ratio*[b] *(95% confidence interval)* |
|---|---|---|---|
| | *Cases* | *Controls* | |
| Common cold[a] | 45/108 (41.7%) | 69/206 (33.5%) | 1.4 (0.8–2.2) |
| Asthma | 7/120 (5.8%) | 13/239 (5.4%) | 1.2 (0.5–3.2) |
| Hayfever | 11/120 (9.2%) | 30/239 (12.5%) | 0.8 (0.4–1.6) |
| Asthma or hayfever | 15/120 (12.5%) | 40/239 (16.7%) | 0.8 (0.4–1.5) |
| Headache | 70/120 (58.3%) | 144/239 (60.3%) | 1.0 (0.6–1.7) |
| Migraine | 22/120 (18.3%) | 61/239 (25.5%) | 0.7 (0.4–1.2) |

[a]Colds: positive is $<1$ cold/yr for at least the last 10 yrs.; [b]Adjusted for age, sex; Colds: less than 1 per yr *vs*. 1 or more per yr (reference group); Other ailments: Ever *vs* Never (reference group).

analyses were repeated for the subset of cases and controls interviewed within 3 months of first diagnosis (81 cases and 145 controls); the results were similar to those reported in Table III.

The relative risk of 6 found by Remy *et al.* (1983) contrasts with our relative risk of 1.4. Our case group however differs substantially from theirs. Melanoma was the type of cancer predominating in their case group (60/110), with a further 21 lymphomas and leukemias and only 21 solid tumours. In contrast 90% of our case group had solid tumours.

There have been a number of case-control studies of allergies and cancer. The definition of allergy and the sites of cancer studied differ from study to study. The information in all but one of these studies was obtained (as in ours) by personal interview. Only two studies (McKee *et al.*, 1967; Shapiro *et al.*, 1971) fulfilled the essential requirements that the information should be collected 'blind' and under identical conditions for cases and controls, and that the age distributions of cases and controls should be similar. Neither of these studies find any relationship between allergy and cancer. Our study adds substance to this result. One other study may be methodologically sound (Allegra *et al.*, 1976) and suggests a large negative effect although based on very small numbers; 1/74 cases and 13/86 controls had a history of allergy. The other studies, of which 5 find a statistically significant negative association (Fisherman, 1960; Mackay, 1966; Ure, 1969, Gabriel *et al.*, 1972; Meers, 1973) and one a significant positive association between history of allergy and cancer (Logan & Saker, 1955), are uninterpretable. Three cohort studies have looked specifically at asthma. Alderson (1974) followed up a large cohort of attenders at a special asthma clinic. Forty three deaths from cancer (excluding lung cancer) had occurred compared to 65.8 expected using England and Wales rates corrected for differences between local and national mortality levels. This study is, however, difficult to interpret. Attenders at a special asthma clinic may be a selected group (possibly of relatively high social class). Their mortality from causes other than cancer was very high (554 observed against 351.2 expected) and it is possible that cancer was recorded as the underlying cause of death less often in these patients than it would be in the general population. Robinette & Fraumeni (1978) studied World War II veterans using Department of Defense and Veterans Administration records. They identified 9550 men hospitalized with bronchial asthma and used as a control group men hospitalized with acute nasopharyngitis. As in Alderson's (1974) study, all cause mortality was high in asthmatics (relative risk 1.7). The relative risk for all malignant neoplasms was 1.3 in asthmatics compared to the control group. This excess was mainly confined to lung cancer and pancreatic cancer. Polednak (1975), in his cohort study of Harvard University graduates, found no difference in cancer mortality between those who did or did not suffer from asthma. The cancer death rate in the asthma group was 8.3% (based on 14 deaths) compared to 8.9% (based on 1060 deaths) in the non-asthma group.

A number of studies have indicated that individuals vary in their susceptibility to common colds (Tyrrell, 1965). It has also been shown that immunity increases with age (Lidwell & Williams, 1961), that adults living in households with young children have more colds than those living in totally adult households (Lidwell & Sommerville, 1951), and that the frequency of colds varies with outdoor temperature (Hope-Simpson, 1958). These three factors must be considered in the present study as potential confounding factors. In our study, few (5%) of those interviewed lived in households with young children. Our age range was however wide although the age distribution of cases and controls was similar. It is possible that recall may be affected by the time of year at interview; a recent cold (more likely during the winter months) would be recalled more readily than a cold six months previously if the interview took place during the summer months. An analysis allowing for age and season of interview had a negligible effect on the results.

The mean number of colds per year experienced by our control group was 1.3. Studies of frequency of common colds using diary records or frequent physical examinations have widely differing results. Lidwell & Williams (1961) in their study of office workers suggested an average of 2 colds per year whereas Dingle *et al.* (1953) found 6 episodes of respiratory disease per person per year. Tyrrell (1965) suggests that this latter figure may be inflated by the definition of a common cold used and the fact that many of the families studied contained young children. Thus our figure using a retrospective questionnaire suggests that recall of colds tends to under-estimate the number per year.

All interviewees were asked to describe a typical cold and this description was compared with terms used by the MRC Common Cold Unit (Tyrrell, D., personal communication). All interviewees described the major common cold symptoms, with the exception of one who described a flu-like illness and was omitted.

Case-control studies such as ours which depend on questioning about events in the past can be particularly subject to unconscious interviewer bias. We have attempted to reduce this potential source of bias as much as possible by ensuring that the interviewer was unaware of the precise hypothesis being tested and of the diagnoses of the patients being interviewed. Although in a few instances (e.g. fractures) the diagnosis was obvious, the final diagnosis of cancer was frequently not made until after interview. Great care was also taken in the choice of a suitable control group. Prior to the study it seemed reasonable to expect that the recall of hospital patients with relatively minor conditions (hernias, varicose veins) might differ substantially from that of patients with life-threatening conditions such as cancer. The major control group was therefore composed of patients with similarly life-threatening disorders with a second group of controls with minor ailments. In the event, the results in the two control groups were identical. Moreover, as expected, cases and controls gave similar histories of migraine and headache. It seems unlikely, therefore, that the lack of association between common colds or allergies and cancer found in this study can be explained by any artefact due to study design.

We thank the consultant medical staff, ward sisters, and patients at St Helier Hospital, Carshalton for their help with this study, Dr Ruth Ellman and Dr Radke Bettelheim for reviewing case notes, and Mrs Betty Lloyd for secretarial help. The Institute of Cancer Research receives support from the Cancer Research Campaign and the Medical Research Council.

### References

ALDERSON, M. (1974). Mortality from malignant disease in patients with asthma. *Lancet*, **ii**, 1475.

ALLEGRA, J., LIPTON, A., HARVEY, H. & 7 others (1976). Decreased prevalence of immediate hypersensitivity (atopy) in a cancer population. *Cancer Res.*, **36**, 3225.

DINGLE, J.H., BADGER, G.F., FELLER, A.E. & 3 others (1953). A study of illness in a group of Cleveland families. *Am. J. Hyg.*, **58**, 16.

FISHERMAN, E.W. (1960). Does allergic diathesis influence malignancy? *J. Allergy*, **31**, 74.

GABRIEL, R., DUDLEY, B.M. & ALEXANDER, W.D. (1972). Lung cancer and allergy. *Br. J. Clin. Pract.*, **26**, 202.

HOPE-SIMPSON, R.E. (1958). Discussion on the common cold. *Practitioner*, **180**, 356.

LIDWELL, O.M. & SOMMERVILLE, T. (1951). Observations on the incidence and distribution of the common cold in a rural community during 1948 and 1949. *J. Hyg.* (*Camb*), **49**, 24.

LIDWELL, O.M. & WILLIAMS, R.E.O. (1961). The epidemiology of the common cold I. *J. Hyg.* (*Camb*), **59**, 309.

LOGAN, J. & SAKER, D. (1955). The incidence of allergic disorders in cancer. *N.Z. Med. J.*, **52**, 210.

MACKAY, W.D. (1966). The incidence of allergic disorders and cancer. *Br. J. Cancer*, **20**, 434.

McKEE, W.D., ARNOLD, C.A. & PERLMAN, M.D. (1967). A double-blind study of the comparative incidence of malignancy and allergy. *J. Allergy*, **39**, 294.

MEERS, P.D. (1973). Allergy and Cancer. *Lancet*, **i**, 884.

POLEDNAK, A.P. (1975). Asthma and cancer mortality. *Lancet*, **ii**, 1147.

REMY, A., HAMMERSCHMID, K., ZANKER, K.S. & 3 others (1983). Lower frequency of infections in cancer patients: do infections protect against cancer? *J. Exp. Clin. Cancer Res.*, **2**, 49.

ROBINETTE, C.D. & FRAUMENI, J.F. (1978). Asthma and subsequent mortality in World War II veterans. *J. Chron. Dis.*, **31**, 619.

SHAPIRO, S., HEINONEN, O.P. & SISKIND, V. (1971). Cancer and allergy. *Cancer*, **28**, 396.

TYRRELL, D.A.J. (1965). *Common colds and related diseases*. Edward Arnold (Pub) Ltd., London.

URE, D.M.J. (1969). Negative association between allergy and cancer. *Scot. Med. J.*, **14**, 51.


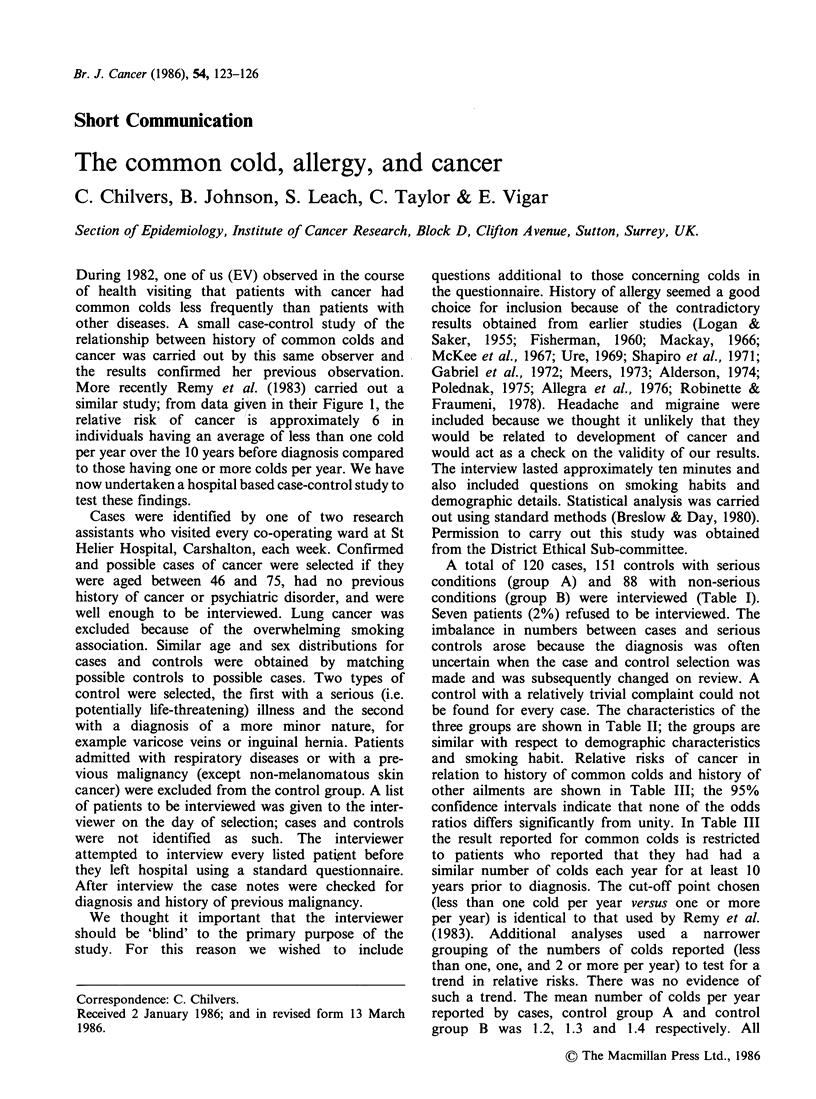

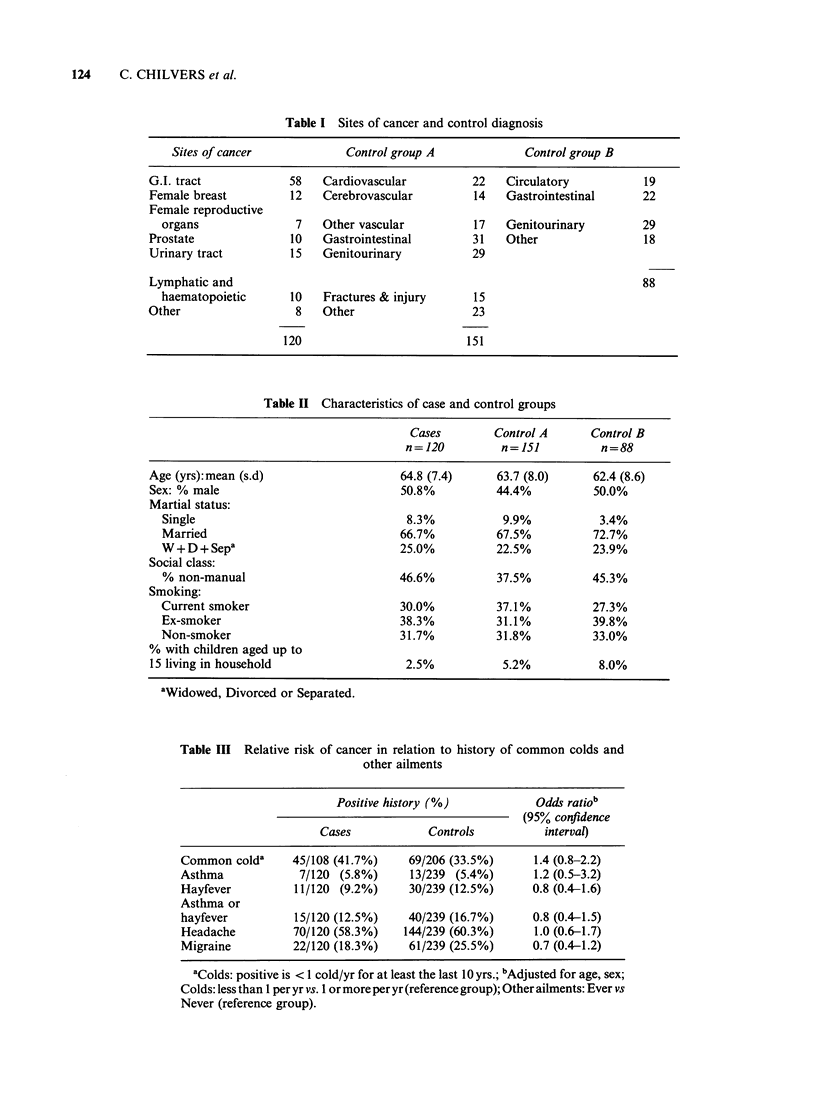

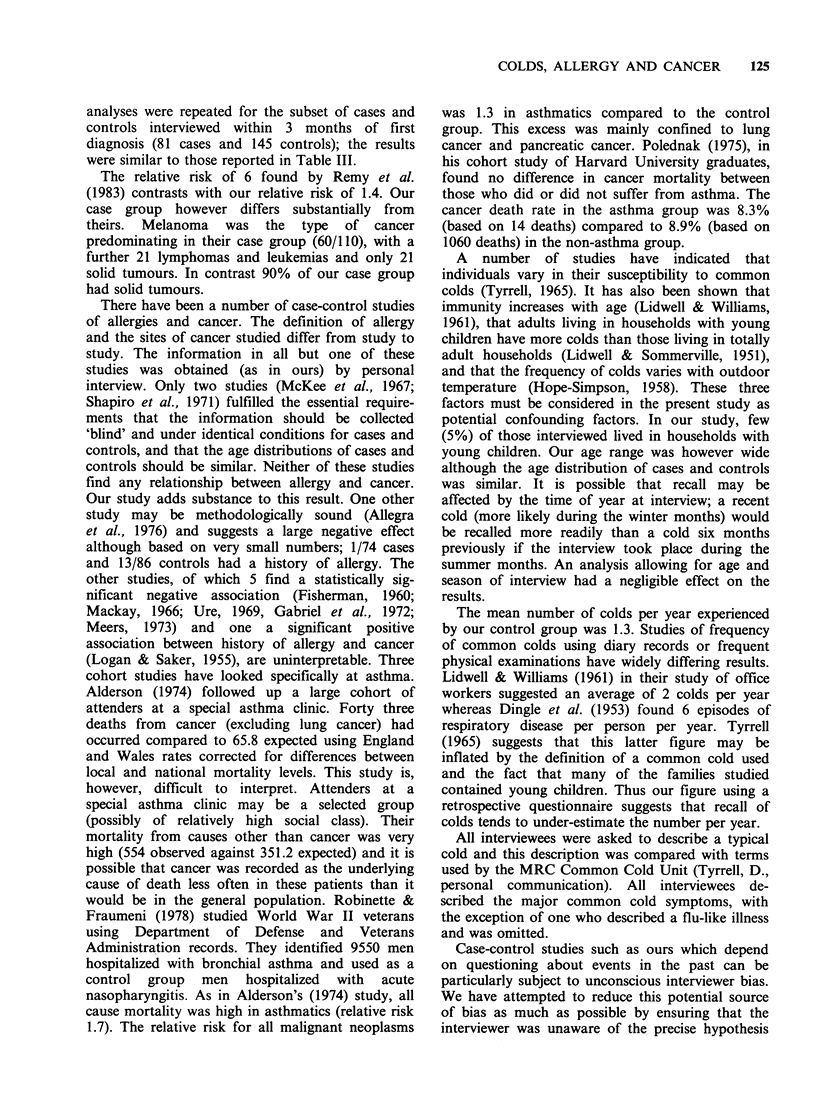

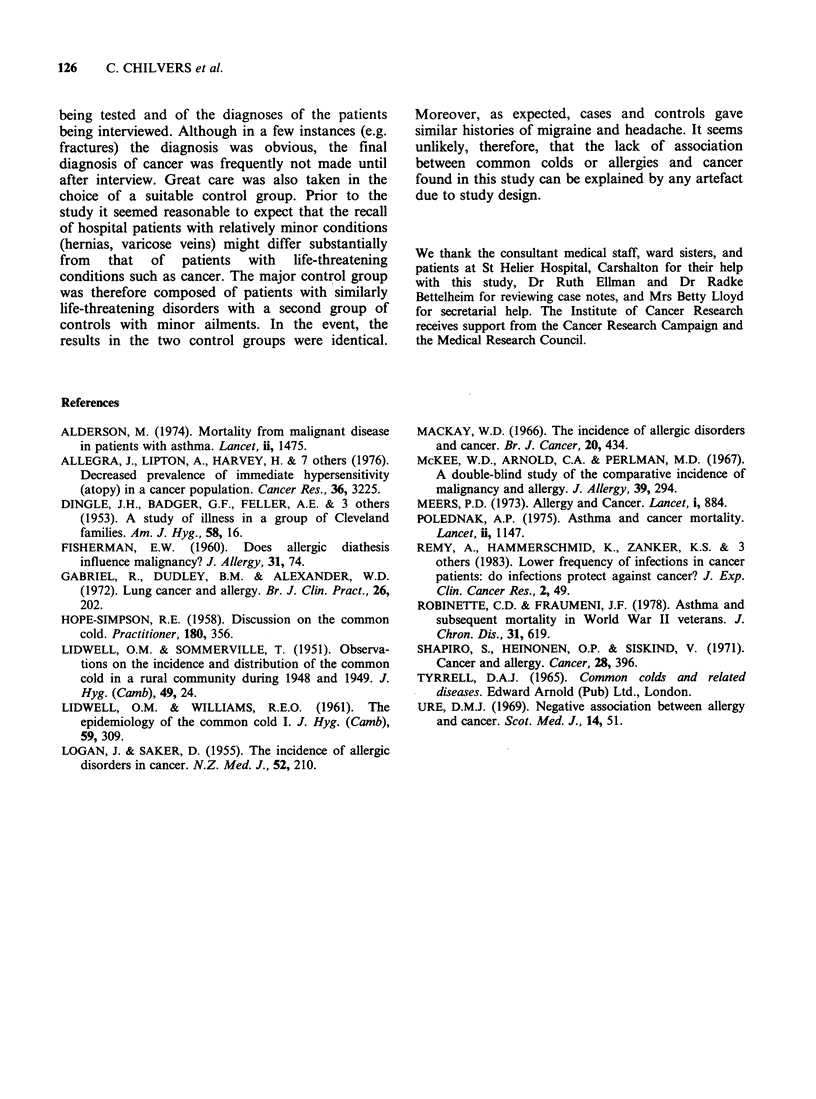


## References

[OCR_00345] Alderson M. (1974). Mortality from malignant disease in patients with asthma.. Lancet.

[OCR_00349] Allegra J., Lipton A., Harvey H., Luderer J., Brenner D., Mortel R., Demers L., Gillin M., White D., Trautlein J. (1976). Decreased prevalence of immediate hypersensitivity (atopy) in a cancer population.. Cancer Res.

[OCR_00359] FISHERMAN E. W. (1960). Does the allergic diathesis influence malignancy?. J Allergy.

[OCR_00363] Gabriel R., Dudley B. M., Alexander W. D. (1972). Lung cancer and allergy.. Br J Clin Pract.

[OCR_00378] LIDWELL O. M., WILLIAMS R. E. (1961). The epidemiology of the common cold. I.. J Hyg (Lond).

[OCR_00383] LOGAN J., SAKER D. (1953). The incidence of allergic disorders in cancer.. N Z Med J.

[OCR_00387] Mackay W. D. (1966). The incidence of allergic disorders and cancer.. Br J Cancer.

[OCR_00391] McKee W. D., Arnold C. A., Perlman M. D. (1967). A double-blind study of the comparative incidence of malignancy and allergy.. J Allergy.

[OCR_00396] Meers P. D. (1973). Allergy and cancer.. Lancet.

[OCR_00398] Polednak A. P. (1975). Letter: Asthma and cancer mortality.. Lancet.

[OCR_00408] Robinette C. D., Fraumeni J. F. (1978). Asthma and subsequent mortality in World War II veterans.. J Chronic Dis.

[OCR_00413] Shapiro S., Heinonen O. P., Siskind V. (1971). Cancer and allergy.. Cancer.

[OCR_00421] Ure D. M. (1969). Negative assoication between allergy and cancer.. Scott Med J.

